# American Dental Convention

**Published:** 1874-09

**Authors:** 


### AMERICAN DENTAL CONVENTION.

This body held its annual session at Saratoga, on the 11th, 12th and 13th of August.

The attendance was quite as good as usual. The subjects presented for consideration were of a practical nature, and the discusssions profitable.

We had the pleasure of meeting a number of the older members of the profession whom we had not seen for a number of years, this gave additional interest to the occasion. The Convention, though not possessing the prestige and influence it once had, is certainly doing good, and especially for those who avail themselves of its meetings.

